# Supplementary material for: Social inequalities in the misbelief of chloroquine’s protective effect against COVID-19: results from the EPICOVID-19 study in Brazil
Source: PLoS One. 2026 Mar 23;21(3):e0341666. doi: 10.1371/journal.pone.0341666 (PMC13008245; doi:10.1371/journal.pone.0341666)
Supplement: S1 Table — aJeopardy index: Zero = male, White, highest education level, and highest wealth quartile; Eight = woman, Black-Brown-East Asian-Indigenous, lowest education level, and lowest wealth quartile. (DOCX) [file pone.0341666.s001.docx]

| Jeopardy index^a^ |  | **n (%)** |
| --- | --- | --- |
| 0 |  | 2,161 (2.9) |
| 1 |  | 6,832 (9.2) |
| 2 |  | 9,387 (12.7) |
| 3 |  | 10,794 (14.6) |
| 4 |  | 12,034 (16.3) |
| 5 |  | 11,944 (16.1) |
| 6 |  | 10,613 (14.3) |
| 7 |  | 7,260 (9.8) |
| 8 |  | 3,052 (4.1) |
