# Supplementary material for: Social inequalities in the misbelief of chloroquine’s protective effect against COVID-19: results from the EPICOVID-19 study in Brazil
Source: PLoS One. 2026 Mar 23;21(3):e0341666. doi: 10.1371/journal.pone.0341666 (PMC13008245; doi:10.1371/journal.pone.0341666)
Supplement: S2 Table — aJeopardy index: Zero = male, White, highest education level, and highest wealth quartile; Eight = woman, Black-Brown-East Asian-Indigenous, lowest education level, and lowest wealth quartile. (DOCX) [file pone.0341666.s002.docx]

| Variables | Total | Jeopardy index^a^ | | | | | | | | |
| --- | --- | --- | --- | --- | --- | --- | --- | --- | --- | --- |
| Sex |  | 0 | 1 | 2 | 3 | 4 | 5 | 6 | 7 | 8 |
| Male | 40.5 | 100.0 | 54.3 | 45.5 | 43.8 | 40.8 | 37.9 | 32.5 | 30.5 | 0.0 |
| Female | 59.5 | 0.0 | 45.7 | 54.5 | 56.2 | 59.2 | 62.1 | 67.5 | 69.5 | 100.0 |
|  |  |  |  |  |  |  |  |  |  |  |
| Race and ethnicity |  |  |  |  |  |  |  |  |  |  |
| White | 37.7 | 100.0 | 77.7 | 51.3 | 40.4 | 34.8 | 27.2 | 23.8 | 18.3 | 0.0 |
| Brown | 44.8 | 0.0 | 17.4 | 36.7 | 43.7 | 47.9 | 52.6 | 54.1 | 55.6 | 66.2 |
| Black | 13.3 | 0.0 | 3.7 | 8.8 | 12.2 | 13.3 | 15.5 | 16.8 | 19.6 | 25.5 |
| East Asian | 2.8 | 0.0 | 0.8 | 2.6 | 2.9 | 2.8 | 3.0 | 3.4 | 3.8 | 4.8 |
| Indigenous | 1.4 | 0.0 | 0.4 | 0.6 | 0.8 | 1.2 | 1.7 | 1.9 | 2.7 | 3.5 |
|  |  |  |  |  |  |  |  |  |  |  |
| Education level |  |  |  |  |  |  |  |  |  |  |
| Higher education | 22.7 | 100.0 | 79.3 | 49.5 | 25.5 | 12.0 | 3.2 | 0.0 | 0.0 | 0.0 |
| High school | 37.5 | 0.0 | 20.7 | 47.0 | 59.6 | 56.8 | 48.8 | 27.1 | 0.0 | 0.0 |
| Elementary school | 17.3 | 0.0 | 0.0 | 3.5 | 12.1 | 20.2 | 26.7 | 34.3 | 26.3 | 0.0 |
| Incomplete elementary school | 22.5 | 0.0 | 0.0 | 0.0 | 2.8 | 11.0 | 21.3 | 38.6 | 73.7 | 100.0 |
|  |  |  |  |  |  |  |  |  |  |  |
| Wealth quartiles |  |  |  |  |  |  |  |  |  |  |
| 1 (Richest) | 25.0 | 100.0 | 88.7 | 60.3 | 26.5 | 10.7 | 4.1 | 0.0 | 0.0 | 0.0 |
| 2^nd^ | 25.0 | 0.0 | 11.3 | 36.6 | 55.6 | 39.9 | 20.2 | 10.1 | 0.0 | 0.0 |
| 3^rd^ | 25.0 | 0.0 | 0.0 | 3.1 | 17.1 | 42.4 | 48.3 | 34.9 | 24.9 | 0.0 |
| 4 (Poorest) | 25.0 | 0.0 | 0.0 | 0.0 | 0.8 | 7.0 | 27.4 | 55.0 | 75.1 | 100.0 |
|  |  |  |  |  |  |  |  |  |  |  |
