# Supplementary material for: Social inequalities in the misbelief of chloroquine’s protective effect against COVID-19: results from the EPICOVID-19 study in Brazil
Source: PLoS One. 2026 Mar 23;21(3):e0341666. doi: 10.1371/journal.pone.0341666 (PMC13008245; doi:10.1371/journal.pone.0341666)
Supplement: S3 Table — Statistically significant associations were highlighted in bold. a Multinomial logistic regression model. Odds ratios indicate the odds of reporting “yes” or “I don’t know” instead of “No”. OR <1 indicates that the participants were less likely, and OR >1 suggests that they were more likely to respond “Yes” or “I don’t know”, instead of “No”, to the question “Do you believe chloroquine offers protection against the coronavirus?” b Jeopardy index: Zero = male, White, highest education level, and highest wealth quartile; Eight = woman, Black-Brown-East Asian-Asian-Indigenous, lowest education level, and lowest wealth quartile. c Slope index of inequality (SII) and Concentration Index (CIX) (values between −100 and 100) according to the jeopardy index. (DOCX) [file pone.0341666.s003.docx]

| **Jeopardy index ^b^** |  | **Round 1 (n=20,709)** | | | **Round 2 (n=25,712)** | | | **Round 3 (n=27,656)** | | |
| --- | --- | --- | --- | --- | --- | --- | --- | --- | --- | --- |
|  |  | **Chloroquine protective effect**  **(Reference: No) ^a^** | | | **Chloroquine protective effect**  **(Reference: No) ^a^** | | | **Chloroquine protective effect**  **(Reference: No) ^a^** | | |
|  |  | **Yes**  **OR (CI95%)** |  | **Don’t know**  **OR (CI95%)** | **Yes**  **OR (CI95%)** |  | **Don’t know**  **OR (CI95%)** | **Yes**  **OR (CI95%)** |  | **Don’t know**  **OR (CI95%)** |
| 0 |  | Ref |  | Ref | Ref |  | Ref | Ref |  | Ref |
| 1 |  | 0.90 (0.71; 1.14) |  | 0.95 (0.74; 1.24) | 0.93 (0.74; 1.18) |  | 0.97 (0.76; 1.23) | 1.00 (0.80; 1.24) |  | **1.23 (1.01; 1.50)** |
| 2 |  | 1.01 (0.81; 1.27) |  | 1.23 (0.97; 1.56) | 0.99 (0.80; 1.24) |  | 1.13 (0.88; 1.43) | 1.18 (0.97; 1.45) |  | **1.37 (1.13; 1.66)** |
| 3 |  | 1.20 (0.97; 1.48) |  | **1.41 (1.12; 1.78)** | 1.11 (0.89; 1.37) |  | **1.52 (1.21; 1.91)** | **1.29 (1.05; 1.59)** |  | **1.81 (1.51; 2.18)** |
| 4 |  | 1.21 (0.98; 1.50) |  | **1.63 (1.30; 2.05)** | 1.17 (0.93; 1.46) |  | **1.58 (1.26; 1.98)** | **1.39 (1.14; 1.69)** |  | **1.93 (1.59; 2.33)** |
| 5 |  | **1.35 (1.09; 1.66)** |  | **1.87 (1.49; 2.35)** | 1.20 (0.96; 1.50) |  | **1.75 (1.41; 2.17)** | **1.53 (1.24; 1.88)** |  | **2.19 (1.82; 2.63)** |
| 6 |  | **1.46 (1.17; 1.83)** |  | **2.14 (1.69; 2.72)** | **1.30 (1.04; 1.63)** |  | **2.13 (1.70; 2.68)** | **1.71 (1.40; 2.10)** |  | **2.49 (2.05; 3.03)** |
| 7 |  | **1.56 (1.25; 1.95)** |  | **2.19 (1.73; 2.76)** | **1.54 (1.21; 1.96)** |  | **2.36 (1.86; 3.00)** | **1.89 (1.52; 2.37)** |  | **2.97 (2.43; 3.63)** |
| 8 |  | **1.55 (1.18; 2.05)** |  | **2.38 (1.83; 3.11)** | **1.50 (1.18; 1.90)** |  | **2.47 (1.89; 3.23)** | **2.09 (1.64; 2.65)** |  | **2.84 (2.26; 3.55)** |
|  |  |  |  |  |  |  |  |  |  |  |
| SII (95%CI) |  | **-4.0 (-6.0; -2.0)** |  | **-14.9 (-17.2; -12.5)** | **-2.7 (-4.4; -1.0)** |  | **-15.7 (-17.6; -13.8)** | **-5.9 (-7.7; -4.2)** |  | **-14.7 (-16.5; -13.0)** |
|  |  |  |  |  |  |  |  |  |  |  |
| CIX (95%CI) |  | **-3.2 (-4.8; -1.6)** |  | **-7.1 (-8.5; -5.6)** | **-2.1 (-3.5; -0.1)** |  | **-7.5 (-8.7; -6.3)** | **-4.3 (-5.6; -2.9)** |  | **-6.9 (-8.0; -5.8)** |
