# Supplementary material for: Social inequalities in the misbelief of chloroquine’s protective effect against COVID-19: results from the EPICOVID-19 study in Brazil
Source: PLoS One. 2026 Mar 23;21(3):e0341666. doi: 10.1371/journal.pone.0341666 (PMC13008245; doi:10.1371/journal.pone.0341666)
Supplement: S4 Table — Statistically significant associations were highlighted in bold. a Jeopardy index: Zero = male, White, highest education level, and highest wealth quartile; Eight = woman, Black-Brown-Indigenous, lowest education level, and lowest wealth quartile. b Jeopardy index: Zero = male, White, highest education level, and highest wealth quartile; Eight = woman, Black-Brown, lowest education level, and lowest wealth quartile. c Multinomial logistic regression model. Odds ratios indicate the odds of reporting “yes” or “I don’t know” instead of “No”. OR <1 indicates that the participants were less likely, and OR >1 suggests that they were more likely to respond “Yes” or “I don’t know”, instead of “No”, to the question “Do you believe chloroquine offers protection against the coronavirus?”. (DOCX) [file pone.0341666.s004.docx]

| **Jeopardy index** |  | **Jeopardy index (White / Black-Brown-Indigenous for race and ethnicity)^a^** | | | **Jeopardy index (White / Black-Brown for race and ethnicity) ^b^** | | |
| --- | --- | --- | --- | --- | --- | --- | --- |
|  |  | **Chloroquine protective effect**  **(Reference: No)^c^** | | | **Chloroquine protective effect**  **(Reference: No)^c^** | | |
|  |  | **Yes**  **OR (CI95%)** |  | **Don’t know**  **OR (CI95%)** | **Yes**  **OR (CI95%)** |  | **Don’t know**  **OR (CI95%)** |
| 0 |  | Ref |  | Ref | Ref |  | Ref |
| 1 |  | 0.95 (0.83; 1.09) |  | 1.05 (0.91; 1.21) | 0.95 (0.83; 1.09) |  | 1.05 (0.91; 1.22) |
| 2 |  | 1.07 (0.95; 1.21) |  | 1.25 (1.09; 1.43) | 1.07 (0.94; 1.21) |  | 1.25 (1.09; 1.43) |
| 3 |  | **1.20 (1.06; 1.35)** |  | **1.59 (1.40; 1.80)** | **1.19 (1.05; 1.35)** |  | **1.58 (1.40; 1.80)** |
| 4 |  | **1.26 (1.11; 1.42)** |  | **1.71 (1.51; 1.93)** | **1.25 (1.11; 1.41)** |  | **1.71 (1.51; 1.93)** |
| 5 |  | **1.36 (1.20; 1.54)** |  | **1.94 (1.73; 2.18)** | **1.36 (1.20; 1.54)** |  | **1.94 (1.73; 2.19)** |
| 6 |  | **1.48 (1.31; 1.68)** |  | **2.24 (1.97; 2.54)** | **1.48 (1.31; 1.68)** |  | **2.24 (1.97; 2.55)** |
| 7 |  | **1.66 (1.45; 1.89)** |  | **2.51 (2.21; 2.84)** | **1.64 (1.43; 1.87)** |  | **2.52 (2.22; 2.85)** |
| 8 |  | **1.73 (1.51; 1.99)** |  | **2.61 (2.24; 3.04)** | **1.70 (1.48; 1.95)** |  | **2.58 (2.21; 3.00)** |
