# Supplementary material for: Social inequalities in the misbelief of chloroquine’s protective effect against COVID-19: results from the EPICOVID-19 study in Brazil
Source: PLoS One. 2026 Mar 23;21(3):e0341666. doi: 10.1371/journal.pone.0341666 (PMC13008245; doi:10.1371/journal.pone.0341666)
Supplement: S5 Table — Jeopardy index: Zero = male, White, highest education level, and lowest wealth quartile; Eight = woman, Black-Brown-East Asian-Indigenous, lowest education level, and lowest wealth quartile. (DOCX) [file pone.0341666.s005.docx]

| Non-pharmacological measures | Jeopardy index | | | | | | | | |
| --- | --- | --- | --- | --- | --- | --- | --- | --- | --- |
|  | 0 | 1 | 2 | 3 | 4 | 5 | 6 | 7 | 8 |
| **Mask use** |  |  |  |  |  |  |  |  |  |
| Yes | 97.6 | 97.8 | 98.1 | 97.8 | 97.8 | 97.8 | 97.9 | 97.8 | 97.5 |
| Don’t know | 0.1 | 0.1 | 0.2 | 0.2 | 0.2 | 0.3 | 0.4 | 0.4 | 0.7 |
| **Stay at home** |  |  |  |  |  |  |  |  |  |
| Yes | 95.2 | 95.2 | 95.8 | 95.4 | 95.5 | 94.9 | 94.9 | 94.2 | 94.1 |
| Don’t know | 0.2 | 0.4 | 0.2 | 0.4 | 0.4 | 0.5 | 0.6 | 0.8 | 0.8 |
