# Supplementary material for: Social inequalities in the misbelief of chloroquine’s protective effect against COVID-19: results from the EPICOVID-19 study in Brazil
Source: PLoS One. 2026 Mar 23;21(3):e0341666. doi: 10.1371/journal.pone.0341666 (PMC13008245; doi:10.1371/journal.pone.0341666)
Supplement: S6 Table — Statistically significant associations were highlighted in bold. a Jeopardy index: Zero = male, White, highest education level, and highest wealth quartile; Eight = woman, Black-Brown-East Asian-Indigenous, lowest education level, and lowest wealth quartile. b Logistic regression model. Odds ratios indicate the odds of reporting “I don’t know” compared to “No/Yes”. OR <1 indicates that the participants were less likely, and OR >1 suggests that they were more likely to respond “I don’t know” instead of “Yes or No”, to the question “Do you believe chloroquine offers protection against the coronavirus?”. (DOCX) [file pone.0341666.s006.docx]

| **Jeopardy index^a^** |  | **Chloroquine protective effect**  **(Reference: No/Yes)^b^** |  |
| --- | --- | --- | --- |
|  |  | **Don’t know**  **OR (CI95%)** |  |
| 0 |  | Ref |  |
| 1 |  | 1.07 (0.93; 1.23) |  |
| 2 |  | **1.22 (1.07; 1.40)** |  |
| 3 |  | **1.52 (1.34; 1.72)** |  |
| 4 |  | **1.62 (1.43; 1.83)** |  |
| 5 |  | **1.78 (1.58; 2.01)** |  |
| 6 |  | **2.02 (1.78; 2.30)** |  |
| 7 |  | **2.17 (1.91; 2.46)** |  |
| 8 |  | **2.20 (1.90; 2.54)** |  |
