# Supplementary material for: Social inequalities in the misbelief of chloroquine’s protective effect against COVID-19: results from the EPICOVID-19 study in Brazil
Source: PLoS One. 2026 Mar 23;21(3):e0341666. doi: 10.1371/journal.pone.0341666 (PMC13008245; doi:10.1371/journal.pone.0341666)
Supplement: Attachment — Submitted filename: Reviewer file.pdf [file pone.0341666.s007.pdf]

Reviewer's report

Thank you for the opportunity to review the manuscript titled “Social inequalities in the misbelief of chloroquine’s protective effect against COVID-19: results from the EPICOVID-19 study in Brazil”. This manuscript presents original research examining the socioeconomic inequalities in beliefs regarding chloroquine’s protective effect against COVID-19 in Brazil, using nationally representative data. The topic is timely and relevant, especially in the context of health misinformation, political influence, and public trust during health crises. The study addresses important public health and equity concerns and employs appropriate methodological approaches, including the use of the Slope Index of Inequality (SII) and Concentration Index (CIX).

However, there are several areas that could benefit from further clarification and refinement:

The **abstract and introduction** are clearly written and provide a coherent overview of the research problem, objectives, and context. The background is well-situated in relevant literature, and the rationale for the study is well-articulated. The authors successfully convey the public health relevance of the research question, and the writing is concise and accessible.

Methodology

1. The manuscript introduces a Jeopardy Index based on the aggregation of four sociodemographic variables reflecting social privilege. While the operationalization appears reasonable, the authors should briefly explain why this specific index was used along with relevant references supporting its use.

2. This section should clearly state which statistical software was used for data analysis (e.g., Stata, R, SPSS), including the version.

Results

1. In the sentence summarizing participant’s demographic characteristics, report the exact percentages for race and education as well.
2. Line 156 can be rephrased like this for clarity: “Approximately 47.9% either lacked knowledge about or did not believe that chloroquine has a protective effect against COVID-19.”
3. In Table 1, I recommend including the counts (n) alongside the percentages (%) for each category of the variables.
4. In Table 2, the sample size is reported as (n = 88,772) in the heading, but this should be written as (N = 88,772) to reflect that it refers to the total sample. Please ensure that the notation for sample size (i.e., N for total sample, n for subsamples) is used consistently throughout the manuscript, including tables, figures, and text.
5. In the tables even though confidence intervals for inequality measures and other estimates are provided, it does not clearly indicate which variables or associations are statistically significant. Although readers can infer significance from whether the CIs include zero, it would improve clarity if statistically significant results were explicitly indicated, either in the text or in table footnotes.

6. The interpretation of SII and CIX results in the text could be expanded. Also, consider including key effect estimates (e.g., percentages, odds ratios, and confidence intervals) in the main text where relevant, so readers don't have to rely only on the tables.

Discussion

1. The discussion is bold and well-supported with relevant references. The integration of political context and its influence on public beliefs is especially commendable. However, this section could be strengthened further by expanding the discussion on how socially disadvantaged groups are disproportionately affected in such scenarios. While this is touched upon, giving it more emphasis would enhance the depth and equity focus of the discussion.
