# Supplementary material for: Social inequalities in the misbelief of chloroquine’s protective effect against COVID-19: results from the EPICOVID-19 study in Brazil
Source: PLoS One. 2026 Mar 23;21(3):e0341666. doi: 10.1371/journal.pone.0341666 (PMC13008245; doi:10.1371/journal.pone.0341666)
Supplement: Attachment — Submitted filename: Reviewer file_10.01.26.pdf [file pone.0341666.s008.pdf]

I have reviewed the revised manuscript and find that the authors have addressed the reviewer comments adequately. The revisions have strengthened the manuscript, particularly in terms of methodological clarity and interpretation of results. I do not have any further concerns and recommend the manuscript for publication.
